# Variability in Prices Paid for Hemodialysis by Employer-Sponsored Insurance in the US From 2012 to 2019

**DOI:** 10.1001/jamanetworkopen.2022.0562

**Published:** 2022-02-28

**Authors:** Riley J. League, Paul Eliason, Ryan C. McDevitt, James W. Roberts, Heather Wong

**Affiliations:** 1 Department of Economics, Duke University, Durham, North Carolina; 2 Department of Economics, Brigham Young University, Provo, Utah; 3 Department of Economics, Fuqua School of Business, Duke University, Durham, North Carolina; 4 National Bureau of Economic Research, Cambridge, Massachusetts

## Abstract

This cross-sectional study compares the prices that private insurers vs Medicare paid for hemodialysis in the US from 2012 to 2019.

## Introduction

Recent proposals have sought to limit the amount dialysis clinics charge private payers,^1^ but little is known about the prices that private insurers actually pay for dialysis.^2,3^ In this study, we provide novel evidence on dialysis prices based on claims data for a large national sample of private employer-sponsored insurance carriers.

## Methods

In this cross-sectional study, we analyzed data from the Health Care Cost Institute, which included all medical claims for enrollees in employer-sponsored health insurance plans offered by carriers covering more than 55 million individuals per year from 2012 to 2019. We reported summary statistics for the prices paid for hemodialysis claims at the national and state levels over time. We also compared these prices with the prices paid by Medicare for the same service, considering both Medicare’s base rate and the highest and lowest possible adjusted rates. Details on the construction of these data are available in the eAppendix in the Supplement. This study was approved by the institutional review board of Duke University and followed the Strengthening the Reporting of Observational Studies in Epidemiology (STROBE) reporting guideline. We analyzed the data using Stata, version 16.1 (StataCorp, LLC).

## Results

The data included 1 987 439 claims for hemodialysis sessions from 2012 to 2019. The mean and median prices that private insurers paid for a dialysis session in the sample were $1287 and $1476, respectively. For context, the highest Medicare base rate during the sample period was $240, less than one-sixth the median private price. Even the highest possible rate paid by Medicare after case-mix and geographic adjustments in this period ($1081) was 26.8% lower than the median price paid by private insurers. Furthermore, prices paid by private insurers varied substantially across our sample, with an SD of $584 and an IQR of $737 to $1671. We observed 47 535 (2.4%) claims with prices of more than $2000 and 21 835 (1.1%) claims with prices of more than $3000.

From 2012 to 2019, the median price for dialysis paid by private insurers increased from $1349 to $1655 (22.7% growth). By contrast, the Medicare base rate for dialysis rose 0.3%, and the maximum adjusted Medicare payment rose 1.4% (Figure).

**Figure.**
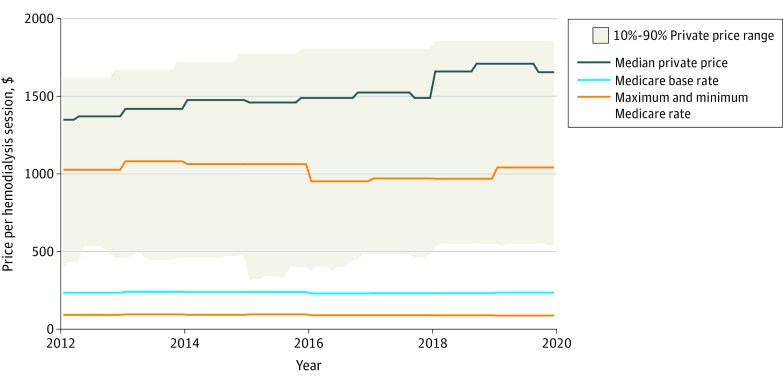
Patterns in Private Insurance Dialysis Prices From 2012 to 2019

The prices paid by private insurers also varied widely across the US. Among the District of Columbia and the 44 states for which we were able to report data, the average price ranged from $950 in Arkansas to $1791 in West Virginia (Table). Our data use agreement prevented us from reporting state-level information based on fewer than 1500 claims or for which the insurer market concentration was high enough that the insurer risked being identified. The 6 states for which we did not report information fell into these categories.

**Table.** Mean and SD of Hemodialysis Prices Paid by Private Insurers by State^a^

Price rank	State	Mean price (SD), $	No. of observations^b^
	National	1287 (584)	1 987 439^b^
1	West Virginia	1791 (200)	4843
2	Rhode Island	1772 (722)	1975
3	Hawaii	1714 (417)	6045
4	South Carolina	1712 (713)	12 678
5	New Hampshire	1667 (351)	5092
6	Oregon	1664 (371)	8299
7	Michigan	1645 (408)	21 564
8	Maine	1640 (403)	7019
9	Alaska	1599 (491)	4226
10	Connecticut	1560 (704)	13 978
11	Iowa	1530 (696)	4233
12	North Carolina	1493 (507)	32 596
13	Virginia	1492 (634)	50 586
14	Nevada	1490 (401)	33 730
15	Minnesota	1481 (471)	5804
16	Mississippi	1478 (498)	10 191
17	Arizona	1444 (539)	55 068
18	Oklahoma	1432 (539)	20 843
19	Ohio	1418 (571)	64 736
20	New Mexico	1414 (721)	3276
21	Delaware	1404 (606)	13 985
22	District of Columbia	1370 (605)	17 039
23	Idaho	1368 (688)	3198
24	Illinois	1331 (584)	73 137
25	Wisconsin	1318 (730)	17 197
26	New Jersey	1309 (521)	106 980
27	Massachusetts	1304 (665)	14 328
28	Georgia	1296 (557)	96 560
29	Texas	1294 (556)	417 723
30	Maryland	1282 (475)	103 494
31	Tennessee	1281 (607)	53 588
32	Colorado	1278 (891)	15 632
33	California	1276 (520)	100 553
34	Indiana	1264 (575)	24 336
35	Washington	1235 (470)	24 471
36	New York	1230 (589)	93 484
37	Pennsylvania	1198 (521)	114 547
38	Missouri	1174 (570)	35 054
39	Nebraska	1161 (602)	4404
40	Florida	1082 (658)	156 482
41	Kansas	1055 (599)	27 352
42	Kentucky	1009 (577)	38 189
43	Utah	1005 (809)	9855
44	Louisiana	960 (535)	39 691
45	Arkansas	950 (677)	3724

^a^ Our data use agreement prevented us from reporting state-level information based on fewer than 1500 claims or for which the insurer market concentration was high enough that the insurer risked being identified. The 6 states for which we did not report information fell in these categories.

^b^ The national observation count is larger than the sum of the state-level observation counts because of the inclusion of data from states for which we could not report state-level data because of the data use agreement.

## Discussion

The prices paid by commercial insurers for dialysis are substantially higher than Medicare’s reimbursements and have increased at a much faster rate during the past decade. This pattern suggests that recent proposals seeking to limit the price of dialysis for individuals with private insurance could bring about large spending reductions,^2^ whereas steering patients from Medicare to private insurance would likely increase spending, a recent concern of policy makers.^4^

A limitation of this study is that although the data used covered more than 30% of the employer-sponsored insurance market,^5^ the results may not represent the prices paid by insurers not in the data set or those paid by private payers in other markets, such as Medicare Advantage or the individual market. Lowering the prices paid by private insurers to Medicare rates and discouraging steering patients onto private plans could bring about substantial savings in spending on hemodialysis.

## Notes

Supplement. **eAppendix.** Details on the Construction of Data Used in the Study
