# Supplementary material for: Variability in Prices Paid for Hemodialysis by Employer-Sponsored Insurance in the US From 2012 to 2019
Source: JAMA Netw Open. 2022 Feb 28;5(2):e220562. doi: 10.1001/jamanetworkopen.2022.0562 (PMC8886517; doi:10.1001/jamanetworkopen.2022.0562)
Supplement: Supplement. — eAppendix. Details on the Construction of Data Used in the Study [file jamanetwopen-e220562-s001.pdf]

Supplementary Online Content

League RJ, Eliason P, McDevitt RC, Roberts JW, Wong H. Variability in prices paid for hemodialysis by employer-sponsored insurance in the US from 2012 to 2019. *JAMA Netw Open*. 2022;5(2):e220562. doi:10.1001/jamanetworkopen.2022.0562

eAppendix. Details on the Construction of Data Used in the Study

This supplementary material has been provided by the authors to give readers additional information about their work.

© 2022 League RJ et al. *JAMA Network Open*.

eAppendix. Details on the Construction of Data Used in the Study

To ensure that the prices we observe are for the same service, we restrict our sample to isolate the price for a single standard hemodialysis session. First, we use claims that bill for Current Procedure Terminology code 90999, indicating dialysis service. To capture the price of a single dialysis session, we limit the sample to claim lines that report one unit of service and for which the period of time covered by the claim is one day. For the same reason, we limit the sample to claims with a type of bill code of 721, indicating an admit through discharge claim, and with revenue code 0821, ensuring that our claims are for outpatient hemodialysis. Including only claims with Place of Service code 65 similarly limits our sample to claims from ESRD treatment facilities, rather than hospitals or transplant centers. This further ensures we are capturing only claims for the most similar service possible, including the place the service is rendered.

Because we are interested in the prices negotiated between dialysis providers and private insurers, we eliminate claims for which the price may have been set by a public insurer like Medicare or Medicaid, keeping only claims where a private insurer is the primary payer of the claim. We also exclude claims for those over 65 (the age at which individuals become immediately eligible for Medicare) and restrict our attention to enrollees whose dialysis claims record does not exceed 33 months, because individuals become eligible for Medicare regardless of age 33 months after being diagnosed with ESRD. We further exclude claims that do not have a positive payment amount. To exclude outlier payments, we (i) keep only claims for months in which an enrollee has 14 or fewer dialysis sessions and (ii) trim the top 1% of observed prices. These

© 2022 League RJ et al. *JAMA Network Open*.

restrictions result a sample of 1,987,439 claims for 25,492 patients from 6,111 providers.

Medicare adjusts payments for dialysis services based on patient-level and facility-level characteristics. Patient-level characteristics include age, low body mass index, body surface area, multiple acute and chronic comorbidities, and whether the patient is within 4 months of the onset of dialysis. Facility-level characteristics include whether the facility treats a low volume of dialysis patients, urbanity, and the wage index of the core-based statistical area of the facility. To construct the maximum adjusted Medicare payment, we calculate the adjustment for a patient-facility pair with the characteristics associated with the highest possible adjustment factor and apply this maximum adjustment factor to the Medicare base rate. This corresponds to a patient aged 18-44, with a body mass index below 18.5, pericarditis, bacterial pneumonia, gastrointestinal tract bleeding with hemorrhage, hemolytic anemia with sickle cell anemia, myelodysplastic syndrome, and monoclonal gammopathy treated at a low-volume facility in the Santa Cruz-Watsonville, CA core-based statistical area. While Medicare also adjusts payment based on a patient's body surface area, there is no maximum level to this adjustment, so we exclude it from the analysis. We similarly construct the minimum adjusted Medicare payment, which corresponds to a patient aged 60-69 or 70-79 (depending on the year) without the comorbidities listed above treated at a facility in urban Puerto Rico.

© 2022 League RJ et al. *JAMA Network Open*.

Note that the Medicare payment includes drugs, supplies, and laboratory services that may be paid separately by private insurers. While private payers may also bundle payment for these services together like Medicare, insofar as they pay them separately, our comparison will understate the price difference between Medicare and private payers.

© 2022 League RJ et al. *JAMA Network Open*.
